# Relative Stability of *cis*- and *trans*-Hydrindanones

**DOI:** 10.3390/molecules20011509

**Published:** 2015-01-15

**Authors:** Motoo Tori

**Affiliations:** Faculty of Pharmaceutical Sciences, Tokushima Bunri University, Yamashiro-cho, Tokushima 770-8514, Japan; E-Mail: tori@ph.bunri-u.ac.jp; Tel.: +81-88-602-8462; Fax: +81-88-655-3051

**Keywords:** hydrindanones, isomerization, isomer, stereochemistry, stability, MM2

## Abstract

The relative stabilities of several *cis*- and *trans*-hydrindanones were compared using both isomerization experiments and MM2 calculations. The generally believed rule that *cis*-hydrindanones are more stable than *trans*-isomers is applicable, but is not always true. This review introduces examples, mainly from studies in our laboratory, to explain these facts.

## 1. Introduction

**Scheme 1:**
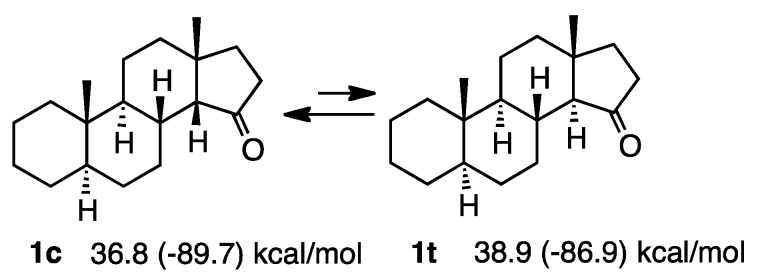
Relative stability of the simple steroid **1**.

Hydrindanones are important intermediates in bioactive natural product synthesis [1,2]. *cis*-Hydrindanones are widely considered to be more stable than the corresponding *trans*-isomers and this is partly because C/D-*trans* steroid ketones at the C-15 position can isomerize to the corresponding *cis* isomers with base treatment [3]. However, the stability of hydrindanones is now known to depend on the ring system. For example, the *trans*-derivative **1t** can be epimerized into the *cis*-derivative **1c** (the suffix “c” denotes *cis* and “t” *trans* for the ring junction) by a simple isomerization reaction. Allinger discussed the relative energies of both *cis* and *trans* isomers calculated by MM2 [3]. Thus, **1c** was more stable than **1t**, as shown in Scheme 1 (the figures in the parentheses are heat of formation values as calculated by MOPAC6). We have been interested in this matter and attempted to determine why *cis*-isomers are so stable. This review describes broad examples of this isomerization and discusses the stability of hydrindanone derivatives, including our own findings.

## 2. Results and Discussion 

### 2.1. Steroid Ketones 

**Scheme 2:**
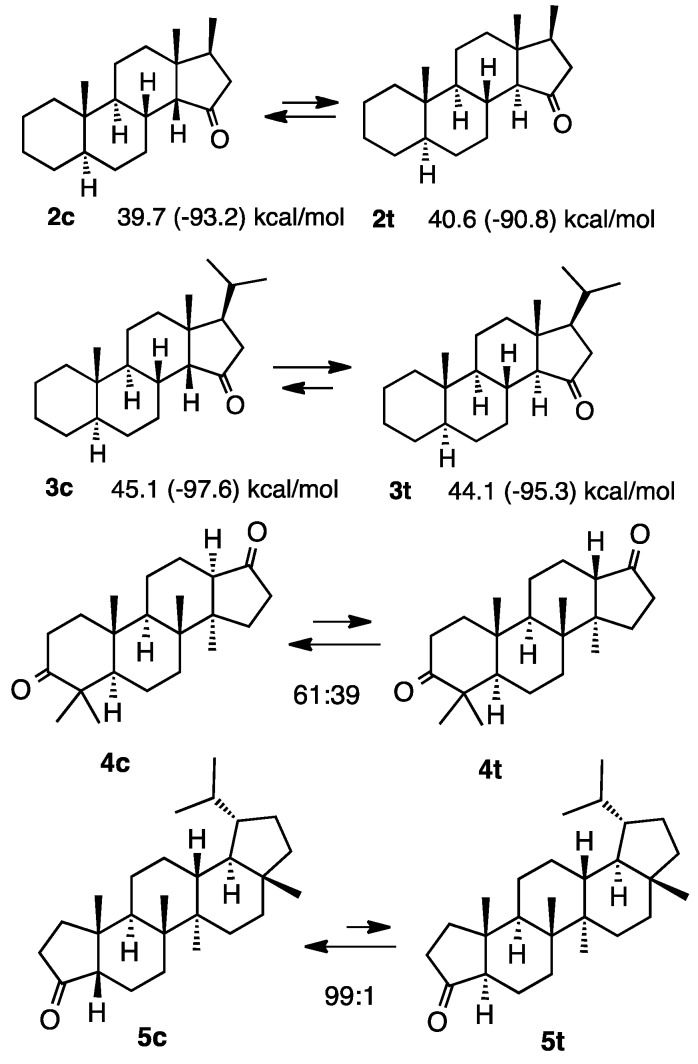
Relative stabilities of simple steroids **2**–**5**.

Two conformations were identified in the *cis*-isomers in rings C and D of steroids, while only a rigid conformation existed in the *trans*-isomer. This was mainly attributed to the methyl group at the juncture position adopting an axial or equatorial orientation in the *cis*-isomer. However, only the axial conformation existed in the *trans*-isomer, which inevitably contributed to its instability. The methylated compound **2c** was more stable than **2t**, demonstrating that the additional methyl group had negligible effect on the relative stability of this system (Scheme 2) [3]. However, the isopropyl group reversed the relative stabilities of **3c** and **3t** because the methyl group of the isopropyl group strongly interacted with the juncture methyl group in the *cis*-isomer **3c**, although the heat of formation of **3c** was less than that of **3t** [3]. The equilibrium ratio was 61:39 in compounds **4c** and **4t**, in favor of the *cis*-isomer **4c** [3]. In the case of compounds **5c** and **5t**, the ratio was 99:1 in equilibrium [3].

### 2.2. Simple 4-Hydrindanones 

**Scheme 3:**
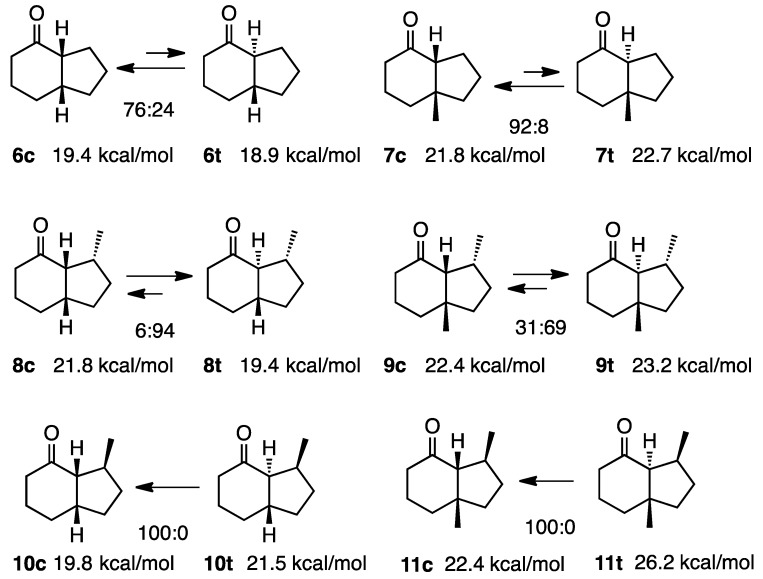
Relative stabilities of *cis*- and *trans*-4-hydrindanones **6**–**11**.

As shown in Scheme 3, several hydrindanones are known to isomerize to *cis*-isomers very easily. Ratios have been determined using base-catalyzed isomerization experiments. Structures have mainly been established by derivatization into hydrazones and X-ray crystallographic analyses. Therefore, the findings obtained appear to be reliable. Thus, we calculated the steric energies of both isomers by MM2 combined with CONFLEX, which was developed by Goto and Osawa to determine out the global minimum conformation [4,5]. The steric energy calculated in this study is shown in Scheme 3; the simplest example, **6c** (19.4 kcal/mol) and **6t** (18.9 kcal/mol), did not follow the calculation (**6c**:**6t** = 76:24) [6]. The ratio of isomerization was reversed to the prediction. This also occurred for compounds **9c** and **9t**. This incompatibility was attributed to non-bonded interactions of substituents including hydrogen atoms with the carbonyl group; however, this has not yet been verified [7].

### 2.3. Dimethyhydrindanones 

**Scheme 4:**
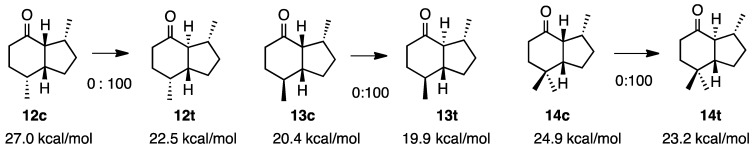
Relative stabilities of *cis*- and *trans*-4-hydrindanones **12**–**14**.

In the course of the total synthesis of isovarelenenol, we examined the relative stabilities of hydrindanones, as depicted in **12c** and **13c** (Scheme 4). Kitagawa reported that the ketone derived from the natural product **12c** very easily isomerized to the *trans*-isomer **12t**; therefore, we avoided acidic or basic conditions in order to examine the side chain of isovarelenenol in more detail [8]. After the successful synthesis of dimethylhydrindanone **12c** by hydrogenation of the hydrindenone, the *cis*-isomer **12c** very easily isomerized to the *trans*-isomer **12t** with a base treatment [9]. Since we had the isomer **13c**, it was also treated with a base to completely isomerize it to **13t**. The MM2 calculation revealed a marked difference in the steric energies of **12c** and **12t** (4.5 kcal/mol). However, this difference was only 0.5 kcal/mol between **13c** and **13t**.

Paquette reported that the *cis* ketone **14c** yielded the *trans*-isomer **14t** with a base treatment [10]. The calculation indicated that there was a 1.7 kcal/mol difference between these ketones, which was understandable because **14c** was similar to **12c** and **13c**.

### 2.4. Isopropylketones 

**Scheme 5:**
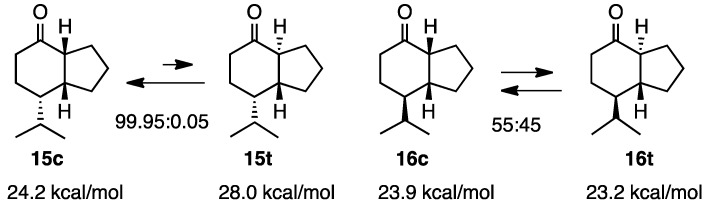
Relative stabilities of *cis*- and *trans*-isopropylketones **15** and **16**.

In the case of **15c**, the *cis* isomer was more stable than the *trans*-isomer **15t**, and this was supported by the MM2 calculation (Scheme 5). However, an almost 1:1 mixture was obtained by the equilibration of **16c** and **16t**. These results were expected by the MM2 calculation revealing only a 0.7 kcal/mol difference [11].

### 2.5. Simple 1-Hydrindanones

**Scheme 6:**
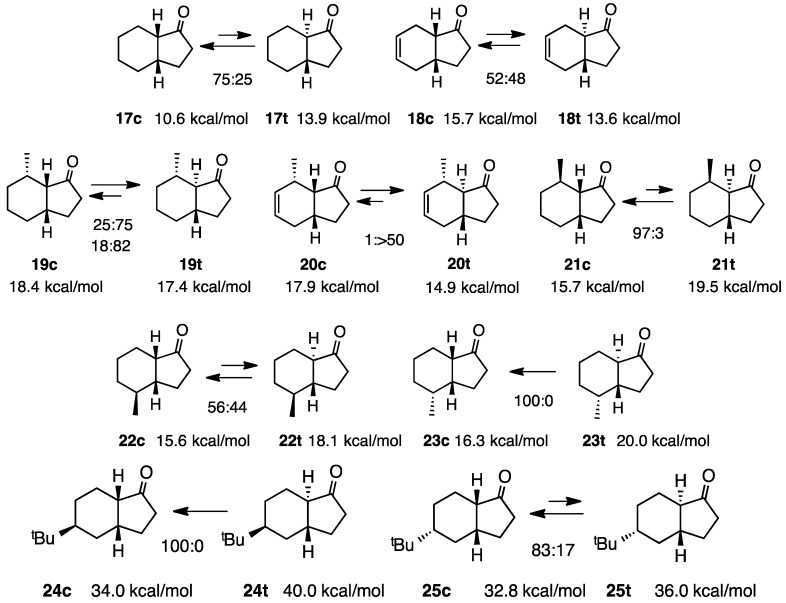
Relative stabilities of *cis*- and *trans*-1-hydrindanones **17**–**25**.

**Scheme 7:**
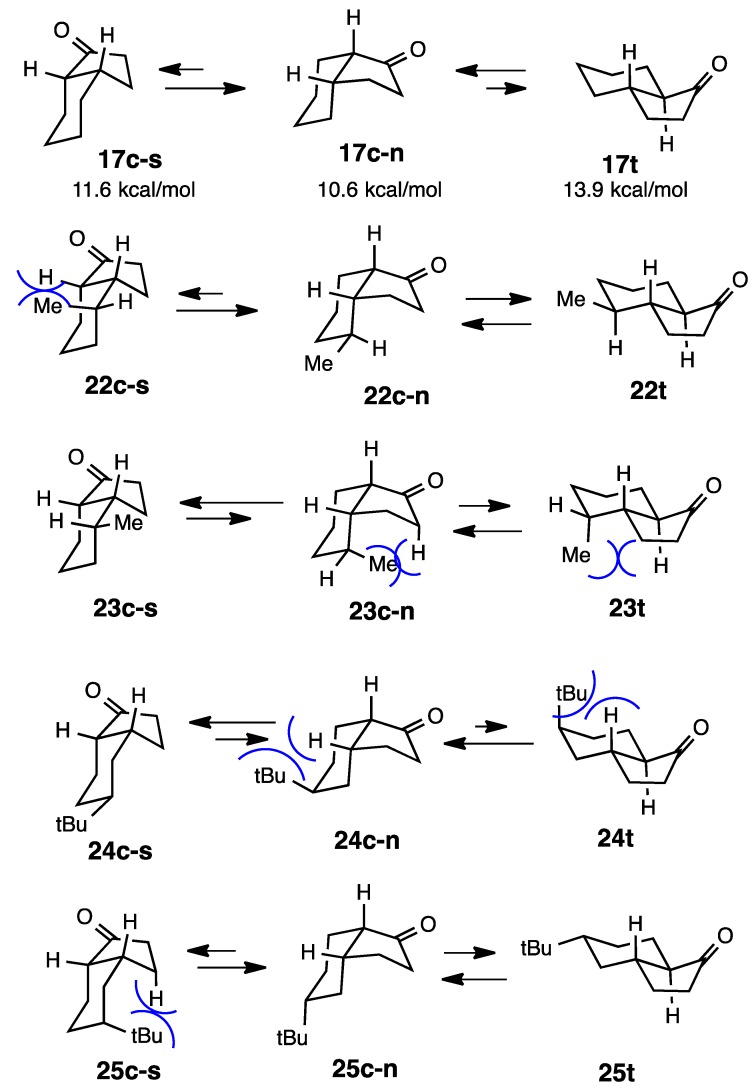
Conformations of compounds **22**-**25**.

In the case of 1-hydrindanones, in which the ketones are in five-membered rings, the simple ketones **17c** and **17t** existed in a ratio of 3:1 in equilibrium; however, the calculation indicated that **17t** was more stable (only 0.4 kcal/mol) [12,13]. *cis*-Hydrindanones generally have two conformations such as **17c-s** and **17c-n** (suffix “s” denotes the steroid form and “n” the non-steroid form) as in the case of *cis*-decalin, while the *trans*-isomer **17t** has only one rigid conformation (s 6 and 7). Therefore, the *cis* isomers exhibit one of two conformations, which is energetically more stable. The calculation indicated that the non-steroid conformation **17c-n** was more stable at 1.0 kcal/mol than **17c-s**. In this case, the calculation fitted the experimental results. The introduction of a double bond to this system pushed the equilibrium to 52:48, although the calculation suggested that the stability of the *trans* isomer **18t** was markedly greater than that of **18c** at 2.1 kcal/mol. The methylated ketones **19** and **20** agreed with the results of the calculation, in favor of *trans*-isomers. There are two studies for ketone **19**, the ratios being 25:75 and 18:82, respectively [13,14]. However, the stability of the diastereoisomer **21c** was markedly greater than that of the corresponding *trans*-isomer **21t** [14]. Other examples of diastereoisomers **22** and **23** show us interesting phenomena (Scheme 6). The compound **23c** was the only isomer that existed in equilibrium, while the ratio for **22c** was 56:44, and the energy difference between these two compounds was 2.5 kcal/mol, in favor of **22c**. This situation was completely reversed in the case of **24** and **25**. Since the *tert*-butyl group always adopts an equatorial conformation in the case of **24t**, the *tert*-butyl group is forced into an axial position and, hence, this isomer cannot exist (**24c**:**24t** = 100:0). This situation can be more clearly understood by the conformations shown in Scheme 7 [14].

### 2.6. 4-Hydroxy-1-hydrindanones 

**Scheme 8:**
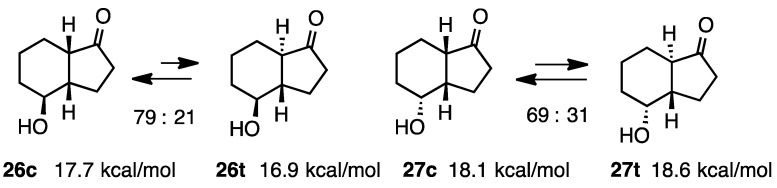
Relative stabilities of *cis*- and *trans*-4-hydroxy-1-hydrindanones **26** and **27**.

**Scheme 9:**
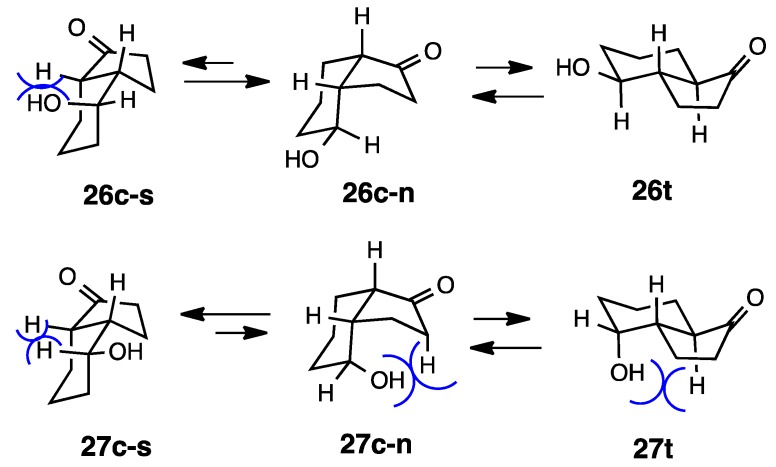
Conformations of *cis*- and *trans*-4-hydroxy-1-hydrindanones **26** and **27**.

We previously described cyclization reactions into hydrindanones using samarium diiodide. The followings are the derivatives prepared by our new methodology [15]. The relative stabilities of these ketones were examined by isomerization with K_2_CO_3_ in MeOH under reflux overnight (both *cis* and *trans* isomers were subjected to the equilibration reaction to get the same ratio). Equilibrium was examined by GC and the structures were established by 2D NMR analyses including NOESY. The calculation of **26c** and **26t** revealed a 0.8 kcal/mol difference (Scheme 8 and Scheme 9). However, base-catalyzed isomerization indicated that the *cis*-isomer was more stable than the *trans*-isomer. This discrepancy was previously encountered in the case of **17** and **18**. The diastereoisomer **27c** was more stable than the *trans*-isomer **27t** at 0.5 kcal/mol and the experimental results showed that the ratio was **27c**:**27t** = 69:31.

**Scheme 10:**
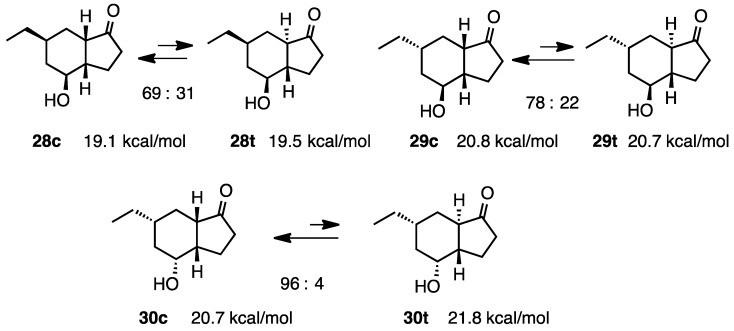
Relative stabilities of *cis*- and *trans*-4-hydroxy-1-hydrindanones **28**–**30**.

**Scheme 11:**
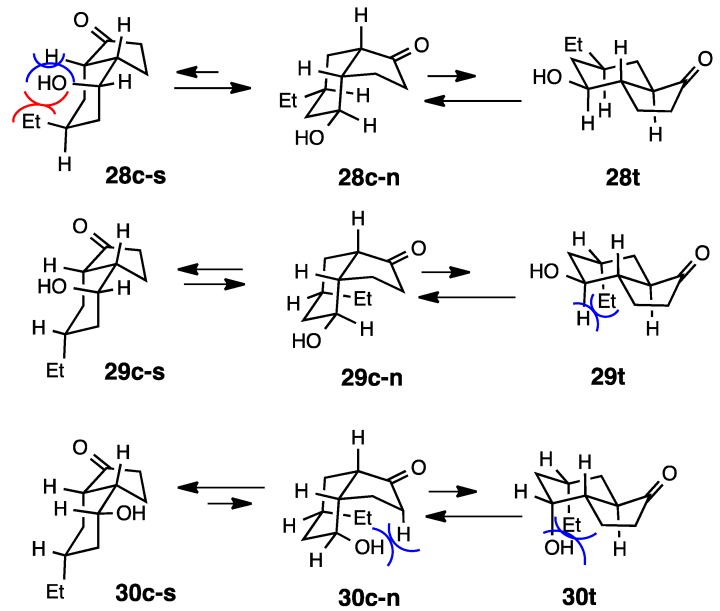
Conformations of *cis*- and *trans*-4-hydroxy-1-hydrindanones **28**–**30**.

The equilibrium *cis*:*trans* ratio for the ethylated derivative **28** was similar to that observed for **26**, with the *cis* epimer predominating (Scheme 10 and Scheme 11) [16]. Whereas the *cis* epimer was computed to be the more stable in **28**, the *trans* epimer **26** was computed to be the more stable in contrast to the observed equilibrium ratio. Although the *cis* epimers were strongly favored at equilibrium in **29** and **30**, very little diffence in energy between the epimers was found by MM2.

**Scheme 12:**
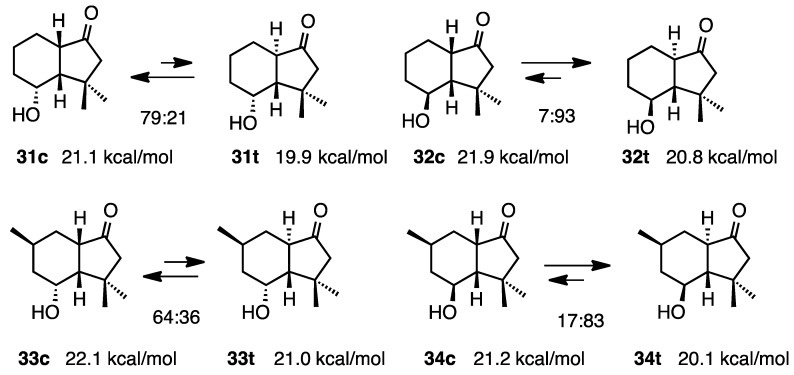
Relative stabilities of *cis*- and *trans*-4-hydroxy-1-hydrindanones **31**–**34**.

If there were two methyl groups at the C-3 position, the compound **31c** did not agree well with the calculation. In contrast, ketone **32t** was more stable than the *cis* isomer **32c**, as was also the case for **34** (Scheme 12) [15].

**Scheme 13:**
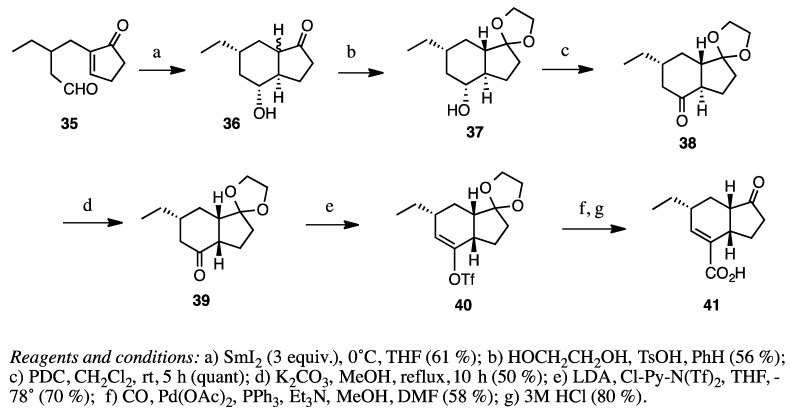
Synthesis of coronafacic acid (**41**).

We succeeded in synthesizing coronafacic acid (**41**) using samarium diiodide-induced reductive cyclization into hydridanones starting from compound **35** (Scheme 13). We used acid or base-catalyzed isomerization twice, as shown in Scheme 13; compound **36** isomerized to the *trans* isomer during acid-catalyzed ketalization and the base-catalyzed isomerization of compound **38** into **39** was achieved. In this synthesis, the initial product mixture of **36** was not purified. However, they all finally converged to the desired isomer [16].

### 2.7. Fused with a Cyclobutane Ring

**Scheme 14:**
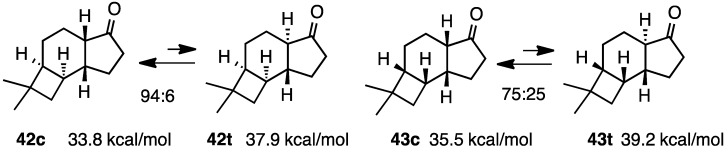
Relative stabilities of *cis*- and *trans*-tricycloketones **42** and **43**.

Denmark* et al.* reported the stability of tricyclic ketones fused with the cyclobutanes, **42c** and **42t**, and **43c** and **43t** (Scheme 14) [17]. Both compounds were predominant in the *cis*-fused isomers at ratios of 94:6 and 75:25, respectively. These values matched the calculated steric energies; the calculated differences were 4.1 and 3.7 kcal/mol, respectively. These compounds appeared to resemble hydrindanones, as shown in Scheme 6.

## 3. Conclusions

The stabilities of *cis*-hydrindanones have been considered to be markedly greater than those of the *trans*-isomers. However, as discussed in this review, some *trans*-hydrindanones are more stable than the corresponding *cis*-isomers, as demonstrated by base-catalyzed isomerization experiments and MM2 calculations (please note that in some cases the latter do not follow the experimental results). The kinds of substituents, the positions and orientations, and thus the conformations, can all affect the stability of the system. Therefore, calculations are advised for the requisite system and base-catalyzed experiments will provide the relevant answers.
